# New insights into the interplay between autophagy and oxidative and endoplasmic reticulum stress in neuronal cell death and survival

**DOI:** 10.3389/fcell.2022.994037

**Published:** 2022-09-16

**Authors:** Yahao Gao, Changshui Wang, Di Jiang, Gang An, Feng Jin, Junchen Zhang, Guangkui Han, Changmeng Cui, Pei Jiang

**Affiliations:** ^1^ Clinical Medical School, Jining Medical University, Jining, China; ^2^ Department of Neurosurgery, Affiliated Hospital of Jining Medical University, Jining, China; ^3^ Cheeloo College of Medicine, Shandong University, Jinan, China; ^4^ Department of Clinical Pharmacy, Jining First People’s Hospital, Jining Medical University, Jining, China

**Keywords:** oxidative stress, autophagy, endoplasmic reticulum stress, neuronal cell death, neuronal cell survival, neuronal cell injury

## Abstract

Autophagy is a dynamic process that maintains the normal homeostasis of cells by digesting and degrading aging proteins and damaged organelles. The effect of autophagy on neural tissue is still a matter of debate. Some authors suggest that autophagy has a protective effect on nerve cells, whereas others suggest that autophagy also induces the death of nerve cells and aggravates nerve injury. In mammals, oxidative stress, autophagy and endoplasmic reticulum stress (ERS) constitute important defense mechanisms to help cells adapt to and survive the stress conditions caused by physiological and pathological stimuli. Under many pathophysiological conditions, oxidative stress, autophagy and ERS are integrated and amplified in cells to promote the progress of diseases. Over the past few decades, oxidative stress, autophagy and ERS and their interactions have been a hot topic in biomedical research. In this review, we summarize recent advances in understanding the interactions between oxidative stress, autophagy and ERS in neuronal cell death and survival.

## 1 Introduction

Autophagy (“self-eating” in Greek) was first described by the Belgian biomedician de Duve in 1963 (Yoshimoto and Ohsumi, 2018). Autophagy is common among eukaryotes such as yeast and mammals and is highly conserved evolutionarily (Levine and Klionsky, 2017). Aged and/or damaged organelles or biological macromolecules are isolated in an engulfing membrane to form autophagosomes, which then combine with lysosomes through a microtubule system to generate autophagy lysosomes in which the enclosed molecules or organelles are degraded (Cao et al., 2021). The main site of autophagosome biosynthesis in neurons is at the axon terminals (Andres-Alonso et al., 2019). Appropriate basic autophagy maintains the homeostasis of nerve cells (Fleming and Rubinsztein, 2020). However, when autophagy in axon terminals is insufficient or transport inhibited, proteins, organelles, and abnormal membrane structures can accumulate in axons, resulting in pathological changes (Yamaguchi et al., 2018). These pathological changes may induce autophagy, enhancing the local biosynthesis of autophagosomes and their accumulation in axons, ultimately leading to cell death (Maday et al., 2012). Autophagy not only protects damaged nerve cells, it can also aggravate nerve cell injury and lead to nerve cell death, which depends primarily on the activity trend and stage of autophagy after brain injury (Duan et al., 2017). Therefore, elucidating the detailed mechanism of autophagy will contribute to the treatment of brain injury and related diseases (Bingol, 2018).

Furthermore, neuroinflammation is associated with the pathogenesis of many neurological disorders including Parkinson’s disease, Alzheimer’s disease, Amyotrophic lateral sclerosis and Huntington disease. Recently, the involvement of autophagy in the regulation of neuroinflammation has drawn substantial scientific interest.

## 2 Interplay between autophagy and oxidative and endoplasmic reticulum stress

### 2.1 Autophagy and oxidative stress

Oxidative stress occurs in the presence of excessive levels of highly active oxidative molecules such as reactive oxygen species (ROS) and reactive nitrogen species. During oxidative stress, the degree of oxidation exceeds the rate of oxide removal, and the resulting imbalance in the oxidation and anti-oxidation systems leads to tissue damage (Bhat et al., 2015). Oxidative stress can directly damage nerve cells through lipid peroxidation (Cornelius et al., 2013), protein oxidation (Hawkins and Davies, 2019), nucleic acid oxidation (Sung et al., 2013), and inflammation (Dandekar et al., 2015). In addition, oxidative stress can adversely affect mitochondria and the nuclear factor–kappa B (NF-κB) and other pathways to indirectly induce neuronal apoptosis (Morgan and Liu, 2011). Other evidence has shown that oxidative stress plays an important role in the pathophysiological processes occurring after brain injury. These effects primarily manifest as an increase in free radical content in brain tissue, the accumulation of peroxide products, and decreases in antioxidants in the brain, including reduced glutathione and superoxide dismutase. This process leads to mitochondrial dysfunction, lipid peroxidation, protein and DNA oxidation, and ultimately neuronal cell death, leaving brain injury patients with lifelong disabilities (Abdul-Muneer et al., 2015).

**FIGURE 1:**
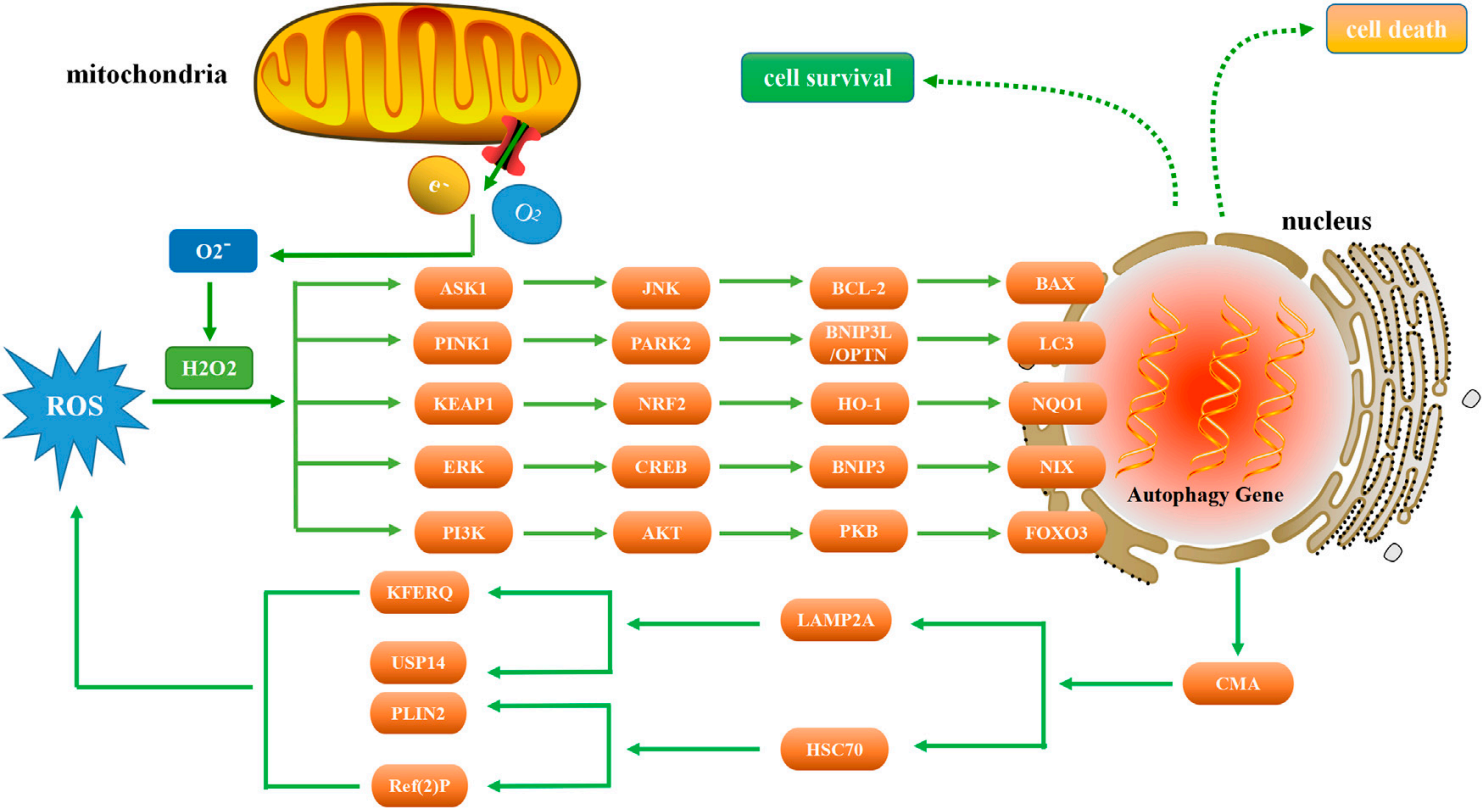
Relationship between autophagy and ROS. As a signaling molecule, ROS regulates autophagy by targeting ASK1, PINK1, KEAP1, ERK, and PI3K. In the presence of excessive ROS, ASK1 and JNK are activated to phosphorylate BCL-2 and increase the expression of BAX, thereby inducing autophagy. PINK1 activation can increase the level of PARK2, ubiquitinate BNIP3L, enhance the production of LC3, and thus induce autophagy. NRF2 is tightly regulated by KEAP1 and induces autophagy through the NRF2-HO-1-NQO1 pathway. When ERK is activated by ROS, autophagy is induced through the ERK-CREB-BNIP3-NIX signaling pathway. Conversely, autophagy suppresses ROS accumulation by eliminating damaged mitochondria via the CMA pathway. ROS regulates the expression of PI3K, and the downstream AKT protein induces autophagy through the AKT-PKB-FOXO3 pathway.

ROS is a general term for a class of molecules or ions with active chemical properties and high oxidation activity (Herb and Schramm, 2021). ROS may be beneficial (oxidative eustress) as signaling molecules, but also detrimental to cells (oxidative stress) when ROS levels are unregulated due to physiological, pathological or pharmacological insults (Scholtes and Giguère, 2021). Substantial evidence indicates that there is a complex relationship between ROS-induced oxidative stress and autophagy (Figure 1).

#### 2.1.1 Role of free radicals in autophagy regulation

Two primary types of ROS have been described: 1) superoxide radicals (O2*-) and hydroxyl radicals (OH-) and other oxygen free radicals, and 2) non-radical derivatives of oxygen, such as hydrogen peroxide (H2O2) (Lushchak, 2014). One of the primary cellular sources of ROS is the mitochondrial inner membrane respiratory chain. Ninety percent of ROS are generated when electrons escape from the mitochondrial electron transport chain (ETC). The ETC is composed of transmembrane protein complexes (I-IV) and ubiquinone and cytochrome c (electron transfer carriers); when these complexes are assembled, together with complex V (F_1_F_0_ATP synthase), the oxidative phosphorylation occurs resulting in ATP production (Villalpando-Rodriguez and Gibson, 2021). Mitochondrial superoxide dismutase 2 (SOD2) is the main component of the ROS processing mechanism in the mitochondrial matrix (Palma et al., 2020). SOD2 is a ubiquitous enzyme in organisms that live in the presence of oxygen, catalyzing the conversion of superoxide into oxygen and H2O2 (Wang et al., 2018), thereby antagonizing and detoxifying ROS. Other proteins that antagonize and detoxify ROS include catalase (Nishikawa et al., 2009), peroxidase (Kidwai et al., 2020), glutathione (Zhang et al., 2020a), and thioredoxin (Ren et al., 2017). Studies have shown that mitochondrial uncoupling plays a role in protecting cells under oxidative stress conditions, including obesity, aging, drug resistance in tumor cells, neurodegeneration, and ischemia-reperfusion damage (Rai et al., 2021). These enzymes are abundant in phagocytes and transfer electrons through protons to generate ROS (Gobert and Wilson, 2017). More importantly, ROS can also be produced in the endoplasmic reticulum and peroxisomes and through the activity of xanthine and endothelial oxidase (Sarniak et al., 2016).

Another important cell source is nicotinamide adenine dinucleotide phosphate oxidase (NOX). NOX is a transmembrane protein specifically involved in generating ROS. Seven subtypes of the NOX family have been identified and characterized: NOX-1, NOX-2 (also known as gp91phox), NOX-3, NOX-4, NOX-5, dual oxidase (DUOX)-1, and DUOX-2 (Chocry and Leloup, 2020). Numerous studies have shown that ROS induce autophagy through NOX. Under different cellular conditions, NOX-derived ROS can regulate autophagy. For example, NOX-2 stimulates autophagy in human macrophages, and the subsequently generated ROS increase the level of LC3-II bound to phagosomes through a toll-like receptor–mediated pathway (Fatima et al., 2021). During *Aspergillus fumigatus* infection, NOX induces macrophage autophagy by generating ROS (Hu et al., 2015). NOX also regulates autophagy in vascular endothelial cells. NOX inhibition was shown to inhibit autophagy in pulmonary artery endothelial cells, resulting in decreased binding of gp91^phox^-p47^phox^ and NOX activity, thus improving pulmonary artery angiogenesis (Teng et al., 2012). NOX-4 is highly expressed in the adventitia and endothelial layers of blood vessels, where it induces ROS-mediated vascular remodeling by regulating autophagy (Barman et al., 2014). In addition, silencing of NOX-2 or NOX-4 has a similar effect on endothelial cell apoptosis and proliferation, possibly due to the common P22^phox^ subunit of both NOX isoforms in ROS-dependent regulation (Djordjevic et al., 2005).

NOX-derived ROS also regulate autophagy in different organs, such as the heart, liver, and lung. NOX is the primary mediator of ROS production in the heart, and NOX-2 deficiency leads to dysplasia of the endocardial cushion and valvoseptal, which is associated with decreased expression of Gata4, Tgfβ2, Bmp2, Bmp4, and Snail1 (Moazzen et al., 2020). NOX-4 plays a major role in mediating autophagy and survival of cardiomyocytes during energy stress by triggering ROS production in the endoplasmic reticulum and stimulating the protein kinase RNA-activated–like endoplasmic reticulum kinase/eukaryotic initiation factor 2α/activating transcription factor 4 (PERK/eIF-2α/ATF4) signaling pathway (Sciarretta et al., 2013). Alamandine induces the migration of hepatic stellate cells and diminishes collagen synthesis by regulating autophagy induced by NOX4-dependent ROS, thereby attenuating hepatic fibrosis (Huang et al., 2020). Moreover, NOX-4 activity correlated with an increase in superoxide formation. NOX-4 forms a complex with p22phox after injury, forming free radicals at neuronal membranes. Alpha-lipoic acid is an oxidative stress inhibitor, which can acutely prevent the development of superoxide, significantly reduce the content of NOX4 and p22phox, and increase the antiapoptotic markers B-cell lymphoma 2 (Bcl-2) and heme oxygenase 1(HO-1) after single blast (Lucke-Wold et al., 2015). Furthermore, the Nox4 pathway produces hydrogen peroxide and superoxide radicals that damage mitochondria, cellular DNA, proteins, and lipid membranes (Lobo et al., 2010). An increase in NOX activity contributes to the increased autophagy in persistent pulmonary hypertension (PPHN)–pulmonary artery endothelial cells (PAECs), whereas the increase of autophagy can reciprocally promote the formation of ROS through NOX-2, leading to impaired angiogenesis involving PPHN-PAECs (Teng et al., 2012). NOX-1/NOX-4 inhibitors significantly reduce levels interferon-α (IFN-α) and IFN-β, thereby attenuating acute lung injury induced by ischemia-reperfusion in mice (Cui et al., 2018).

There is also a significant body of literature indicating that NOS is involved in the regulation of autophagy. In mammals, three distinct genes encode NOS isoenzymes, neuronal (nNOS or NOS-1), inducible (iNOS or NOS-2), and endothelial (eNOS or NOS-3) (Thomas et al., 2008). nNOS is present only in peripheral nerves (Gao et al., 2008), whereas eNOS is present in vascular endothelial cells (Siragusa and Fleming, 2016). iNOS is primarily produced by mesenchymal and parenchymal cells under pathologic conditions following stimulation by endotoxin and cytokines and expressed under transcriptional and post-transcriptional control (Carnovale and Ronco, 2012). NOS-1 prevents excessive autophagy and promotes the survival of nasopharyngeal carcinoma cells through *S*-nitrosylation of phosphatase and tensin homolog (PTEN) and activation of the AKT/mTOR signaling pathway (Zhu et al., 2017). Overexpression of eNOS and iNOS (but not nNOS) in liver cancer tissues contributes to increase NO levels in liver cancer patients, which in turn inhibit autophagy by disrupting the binding of BECN1/VPS34 and promoting apoptosis by increasing the B cell lymphoma-2 (BCL-2)/BECN1 interaction in liver cancer cells (Zhang et al., 2019a). Silica nanoparticles interfere with the NO/NOS system by inhibiting the PI3K/AKT/mTOR pathway, thereby reducing the activity of eNOS and significantly increasing the activity of iNOS, inducing inflammation and activating autophagy, which ultimately leads to endothelial dysfunction (Duan et al., 2014).

#### 2.1.2 Regulation of autophagy by ROS

Existing research data show that ROS plays a crucial role in autophagy activation. Whether it is the result of autophagy survival or death, or the initiation conditions starvation, pathogen or death receptor, ROS is invariably involved in it. However, the nature of such participation remains unclear. Interestingly, although the link between ROS and autophagy has been observed under different pathological conditions, the activation mode of autophagy and its potential protective effect are still not completely clear. This part summarizes the latest internal mechanism of ROS regulating autophagy under relevant pathological conditions.

##### 2.1.2.1 ROS-ASK1-JNK-BCL-2-BAX autophagy

The production of ROS can activate the ASK1 (apoptosis signal-regulating kinase 1) and JNK (c-Jun N-terminal kinase) pathways (Baregamian et al., 2009). Under oxidative stress, ROS induces the dissociation of thioredoxin, a protein that regulates cellular reduction and oxidation (redox), from the N-terminal region of ASK1, which is then activated by the oligomerization and phosphorylation of a key threonine residues, leading to autophagy (Guo et al., 2017). When ROS is generated, it leads to the activation of JNK, phosphorylation of BCL-2, and dissociation of the BECN1/BCL-2 complex (Zhu et al., 2018), and BAX (is a pro-apoptotic gene) increases significantly in a time-dependent manner (Lu et al., 2017). TMBIM6 (transmembrane BAX inhibitor motif containing 6) is a Ca^2+^ channel-like protein known to enhance autophagy by regulating lysosomal calcium (Kim et al., 2021). Inhibiting the expression of p22phox and NOX5 genes can reduce the level of ROS under hypoxia and reduce the occurrence of autophagy. The expression of BAX protein decreased significantly, while the expression of BCL-2 protein increased significantly (He et al., 2021). Vortioxetine can induce apoptosis and autophagy by increasing the levels of BAX, BECN1 and LC3, and by downregulating BCL-2 (Lv et al., 2020). Moreover, ecliptasaponin A enhances autophagy by triggering the ROS/ASK1/JNK pathway, thereby inducing apoptosis in human lung cancer cells (Han et al., 2019). Pristimerin induces apoptosis and autophagy via ROS/ASK1/JNK pathway activation *in vitro* and *in vivo*. And inhibition of ROS with N-acetyl cysteine significantly decreased pristimerin-induced cell death by inhibiting the phosphorylation of ASK1 and JNK (Zhao et al., 2019).

##### 2.1.2.2 ROS-PINK1-PARK2-BNIP3L/OPTN autophagy

Autophagy can be regulated by ROS/PINK1 (PTEN-induced putative kinase 1) signaling pathway. ROS-PINK1-mediated mitophagy plays a protective role in contrast-induced acute kidney injury by reducing NLRP3 (NOD-like receptor family pyrin domain containing 3) inflammasome activation (Lin et al., 2019). BNIP3L (BCL2/adenovirus E1B 19 kDa protein-interacting protein 3L) acts downstream of the PINK1/PARK2 (parkin) pathway to induce mitophagy. Ubiquitination of BNIP3L by PARK2 recruits NBR1 (neighbor of Brca1 gene) into mitochondria and targets mitochondria for degradation (Gao et al., 2015). There is now evidence that NBR1 is dispensable for PARK2 mediated mitophagy (Shi et al., 2015). The PINK1/PARK2/OPTN (optineurin) pathway of mitophagy is activated for protection in septic acute kidney injury (Wang et al., 2021a). Both BNIP3L and OPTN can promote the formation of autophagosomes by enhancing the production of LC3 and the maturation of phagocytes (Bansal et al., 2018; Roperto et al., 2019). Under the stress of high glucose, the production of ROS in cells increased, and the expression levels of PINK1, PARK2, BNIP3L and LC3-II increased significantly, while the expression of LC3-I and the number of autophagosomes decreased significantly (Huang et al., 2018).

##### 2.1.2.3 ROS-KEAP1-NRF2-HO-1-NQO1 autophagy

The increase of ROS induces NRF2 (nuclear factor E2-related factor 2), which leads to autophagy (Xie et al., 2021). Under normal conditions, NRF2 is tightly regulated by the KEAP1 (kelch like ECH associated protein 1) (Imai et al., 2021). Curcumin induced autophagy and alleviated renal oxidative stress through NRF2/HO-1 (Heme oxygenase-**1**) pathway, which significantly alleviated the development of membranous nephropathy (Di Tu et al., 2020). Activation of the ROS/HO-1/NQO1 (NADPH quinone oxidoreductase 1) signaling pathway contributes to the copper-induced oxidative stress and autophagy in duck renal tubular epithelial cells (Fang et al., 2021). In addition, methane alleviates acetaminophen-induced liver injury by inhibiting inflammation, oxidative stress, endoplasmic reticulum stress, and apoptosis through the NRF2/HO-1/NQO1 signaling pathway (Feng et al., 2019). The lack of NQO1 leads to the imbalance of autophagy initiation, which leads to the deterioration of renal cell injury (Hong et al., 2021). Interestingly, NQO1 deficiency can also lead to enhanced autophagy through AMPK/TSC2 (tuberous sclerosis complex 2)/mTOR signaling pathway, thereby aggravating renal injury (Kim et al., 2016).

##### 2.1.2.4 ROS-ERK-CREB-BNIP3-NIX autophagy

As an important intracellular messenger, ROS activates the ERK (extracellular signal-regulated kinase) pathway by stimulating epidermal growth factor (EGF) (Rodrigues et al., 2012) and platelet-derived growth factor (PDGF) (De Minicis et al., 2008) receptors or by directly oxidizing C118 residues in Ras (Deora et al., 2000). Presenilin 1 deficiency suppresses autophagy in human neural stem cells through reducing γ-secretase-independent ERK/CREB (cAMP-response element binding protein) signaling (Chong et al., 2018). Sirt3 regulated Bnip3-related mitophagy via the ERK-CREB signalling pathway; blockade of the ERK-CREB axis repressed mitophagy activity and abrogated Sirt3-mediated mitochondrial protection as well as cell survival (Li et al., 2018). BNIP3 plays a key role in cellular response to stress by regulating apoptosis and autophagy. Overexpression of BNIP3 resulted in up regulation of autophagy related proteins, including LC3, autophagy related protein 7 (ATG7) and BECN1 (Ma et al., 2020). BNIP3 is partially co-localized with autophagosomes, and the interaction between BNIP3 and vimentin, an intermediate filament protein, can regulate autophagy in cells (Liu et al., 2020a). Tanshinone I inhibits metastasis of cancer cells by inducing BNIP3/NIX-mediated mitophagy and reprogramming mitochondrial metabolism (Cui et al., 2022). And BNIP3L/NIX-mediated mitophagy also protects against glucocorticoid-induced synapse defects (Choi et al., 2021). In addition, ROS/ERK pathway can also regulate the downstream component m-TOR to induce autophagy. Furthermore, lactate regulates autophagy through ROS-mediated activation of ERK/m-TOR/p-70S6K pathway in skeletal muscle (Nikooie et al., 2021).

##### 2.1.2.5 ROS- PI3K -AKT -PKB-FOXO3 autophagy

The PI3K (phosphoinositide 3-kinase) pathway played an important role in regulating the autophagic response of cells in response to changing ROS levels. Phosphatase and tensin homolog (PTEN) protein are the negative regulators of the PI3K pathway, and it has proautophagic activities. Targeting PTEN can regulate autophagy and promote the repair of damaged neurons (Yu et al., 2020a). The downstream AKT protein regulates autophagy in response to changes in ROS. AKT plays an important role in autophagy by activating rapamycin complex 1 (mTORC1) and mTORC2. mTORC1 and mTORC2 inhibit autophagy at moderate ROS levels, but mTORC2 can promote cellular senescence by autophagy at high ROS levels (Kma and Baruah, 2022). ROS accumulate downstream of BECN1, and forkhead box O3 (FOXO3) is activated downstream of cellular ROS accumulation (Zhou et al., 2019). FOXO3 rapidly induces autophagy after cytokine deprivation. Moreover, like some other transcription factors, FOXO3 activity involves transactivation of autophagy-related genes (Fitzwalter and Thorburn, 2018). FOXO3 controls the transcription of autophagy-related genes, including LC3 and BNIP3 (Aucello et al., 2009). Activation of the AKT/PKB (protein kinase B) signaling pathway blocks FOXO3 activation and autophagy; although BNIP3 mediates the effect of FOXO3 on autophagy, this effect cannot be prevented by proteasome inhibitors (Mammucari et al., 2007). Dexamethasone (Dex) increases intracellular ROS levels, AKT and FOXO3 expression, and autophagy flux in human chondrocytes via the ROS/AKT/FOXO3 signaling pathway. Silencing FOXO3 leads to down-regulation of Dex-induced autophagy. Moreover, knockdown of FOXO3 was shown to increase Dex-induced apoptosis as well as ROS levels in chondrocytes (Shen et al., 2015). These findings suggest that FOXO3 and LC3 are potential therapeutic targets for certain diseases.

#### 2.1.3 Autophagy regulates ROS formation

In the response to ROS damage, autophagy is essential for the clearance of damaged mitochondria. In particular, the ubiquitin (Ub)-proteasome system (UPS), which eliminates oxidized proteins, is considered the primary mechanism. The majority of misfolded proteins are degraded by the UPS, in which Ub-conjugated substrates are deubiquitinated, unfolded and cleaved into small peptides when passing through the narrow chamber of the proteasome. The substrates that expose a specific degradation signal, the KFERQ sequence motif, can be delivered to and degraded in lysosomes via the CMA. Aggregation-prone substrates resistant to both the UPS and the CMA can be degraded by macroautophagy, in which cargoes are segregated into autophagosomes before degradation by lysosomal hydrolases (Ciechanover and Kwon, 2015).

Known players or pathways that link the UPS to autophagy include the N-terminal arginylation of the N-end rule pathway, the unfolded protein response, and p53. The N-end rule pathway mediates autophagic proteolysis and modulates the activity of the autophagic adaptor p62 as well as autophagosome biogenesis. In this crosstalk, the accumulation of autophagic cargoes such as ubiquitinated proteins and aggregates induces the N-terminal arginylation of ER-resident chaperones such as BiP/GRP78, calreticulin, and protein disulfide isomerase, resulting in the cytosolic accumulation of their arginylated species. Amongst these, arginylated BiP, R-BiP, is associated with misfolded proteins, and its N-terminal Arg binds the ZZ domain of p62. This N-end rule interaction induces a conformational change of p62, exposing its PB1 and LC3-interacting domains, which accelerates its self-oligomerization and targeting to autophagosomes (Cha-Molstad et al., 2017). In the UPS-ER-autophagy circuit, proteasome inhibition causes the excessive accumulation of misfolded proteins in the ER lumen, which in turn leads to the dissociation of the molecular chaperone BiP from the ER membrane receptors PERK, IRE1α and ATF6α. The activated receptors initiate the UPR to temporarily halt global translation and facilitate the synthesis of ER-residing molecular chaperones. These recruited chaperones assist the folding of incoming ER clients and the degradation of terminally misfolded clients through ERAD. In parallel, each of PERK, IRE1α and ATF6α on the ER cytosolic surface activates its own downstream pathways toward autophagy (Hetz et al., 2015). Proteasome inhibition can induce autophagy via the metabolic stabilization of the transcriptional factor p53 (Lagunas-Martínez et al., 2017).

In fact, chaperone-mediated autophagy (CMA) can only degrade cytosolic proteins which contain a CMA-targeting motif, biochemically related to the pentapeptide KFERQ. The CMA pathway degrades and thus regulates specific cell proteins in a timely manner. In the case of chronic nutrient deficiency, the CMA pathway is responsible for the degradation of 30% of cytoplasmic proteins (Dice, 2007). The chaperone heat shock cognate 70 kDa (HSC70) and LAMP-2A are key components of CMA (Losmanová et al., 2020). CMA was the first lysosomal process discovered by which cellular proteins are selectively degraded. The process involves the identification, folding, translocation, and degradation of substrate proteins (Yang et al., 2019). Proteins are the only substrate degraded by this pathway, but not all proteins can be degraded by CMA. To be a CMA substrate, the amino acid sequence of a protein must include a specific targeting motif. This motif binds to HSC70 to bring the substrate protein into the lysosome for degradation (Kaushik and Cuervo, 2018). HSC70 recognizes pentapeptide motifs (KFERQ-like motifs) that are essential for the degradation of CMA substrate proteins in lysosomes (Kirchner et al., 2019). To date, HSC70 remains the only molecular chaperone that has been shown to directly bind to KFERQ-like motifs. KFERQ-like motifs serve a “bait” role to attract HSC70 as a cytoplasmic partner. After HSC70 binds to this region, the protein is targeted for lysosomal degradation (Chiang et al., 1989). In general, HSC70 participates in a variety of cellular functions by promoting the folding of unfolded or misfolded proteins. However, HSC70 also binds KFERQ-like motifs to target proteins for degradation via CMA. HSC70 also participates in the selective degradation of ubiquitin-positive protein aggregates (Arndt et al., 2010). Direct interaction between gastrokine-2 and HSC70 promotes ROS-induced mitochondrial dysfunction, inhibits the NF-κB signaling pathway, and activates the JNK signaling pathway, ultimately leading to an increase in the level of apoptosis (Zhang et al., 2019b). HSC70-based autophagy peptides effectively reduce the levels of Aβ oligomers in cortical neurons in AD patients and protect cortical neurons from Aβ oligomer–induced neurotoxicity (Dou et al., 2020). Dynamic interaction between USP14 (a de-ubiquitination enzyme) and the chaperone HSC70 mediates crosstalk between ERS and autophagy. Regulation of the USP14-HSC70 axis is therefore a potential therapeutic approach for Huntington disease, as it would beneficially affect a variety of protein homeostasis pathways (Srinivasan et al., 2020).

Interestingly, the induction of oxidative stress by CMA can also occur via an increase in the transcription of the LAMP-2A gene (Kiffin et al., 2004), whereas activation of CMA by nutrient deprivation depends on a reduction in LAMP-2A degradation and relocation of the receptor on the lysosomal membrane (Cuervo and Dice, 2000). LAMP-2A was the first lysosomal component required for CMA to be identified (Majeski and Dice, 2004). The level of LAMP-2A in the lysosomal membrane can be increased by reducing its degradation and/or promoting its redistribution from the lysosomal cavity to the lysosomal membrane. LAMP-2A is a transmembrane protein that can translocate proteins to lysosomes (Rout et al., 2014). Transport of LAMP-2A is regulated by cystine, Rab11, and Rab-interacting lysosomal protein, and the treatment of cells in cystinosin deficiency with CMA activator increases the localization of LAMP-2A in lysosomes and increases the survival rate of the cells (Zhang et al., 2017). Expression of DYNC1LI2 (LIC2) preserves LAMP-2A and CMA activity, cell homeostasis, and the localization of LRP2/megalin in cystinotic proximal tubular cells in cystine disease. Decreased expression levels of chaperone heat shock protein A5 (HSPA5)/glucose-regulated protein 78 (GRP78) and the transcription factors ATF4 and DDIT3–C/EBP homologous protein (CHOP) are related to ERS (Rahman et al., 2021). LAMP-2A activation of CMA requires the involvement of p38 MAPK. ER stressors induce PERK-dependent activation and mitogen-activated protein kinase 4 recruitment to lysosomes, in turn activating p38 MAPK at lysosomes. The p38 MAPK directly phosphorylates the CMA receptor LAMP-2A at T211 and T213, resulting in accumulation in the membrane and the induction of active conformational changes that ultimately activate CMA (Li et al., 2017).

The two-domain architecture of the lumenal domains of LAMP-2A mediates the interaction with HSC70 at the cytoplasmic surface of the lysosome (Ikami et al., 2022). In addition to HSC70, other co-molecular chaperones along with LAMP-2A are involved in mediating the translocation of CMA substrates across the lysosomal membrane, such as HSP90 and HSP40 (Agarraberes and Dice, 2001). An increase in sirtuin 3 (an NAD^+^-dependent deacetylase) expression stimulates formation of the LAMP-2A/HSC70/perilipin-2 complex and promotes CMA (Zhang et al., 2020b). A lack of sorting nexin 10 inhibits cathepsin-A maturation and increases the stability of LAMP-2A, leading to increased CMA activity (You et al., 2018). LAMP-2A effectively prevents the accumulation of the autophagy defect marker Ref (Levine and Klionsky, 2017)P/p62 in adult brain under acute oxidative stress. Studies have shown that LAMP-2A can also enhance autophagic flux in the *Drosophila* brain PD model, thereby enhancing anti-stress responses and exerting neuroprotective effects (Issa et al., 2018). In a PD model, geniposide exhibited neuroprotective effects by inhibiting α-synuclein (α-Syn) expression via the miR-21/LAMP-2A axis (Su et al., 2016). The transcription factor NFE2L2/NRF2 (nuclear factor, erythroid 2 like 2) modulates CMA via the regulation of LAMP-2A (Pajares et al., 2018). In addition, as NFE2L2/NRF2 also regulates macroautophagy genes (Pajares et al., 2016), it functions as a regulatory node in the proteolytic network involving macroautophagy and CMA.

### 2.2 Autophagy and ERS

**FIGURE 2:**
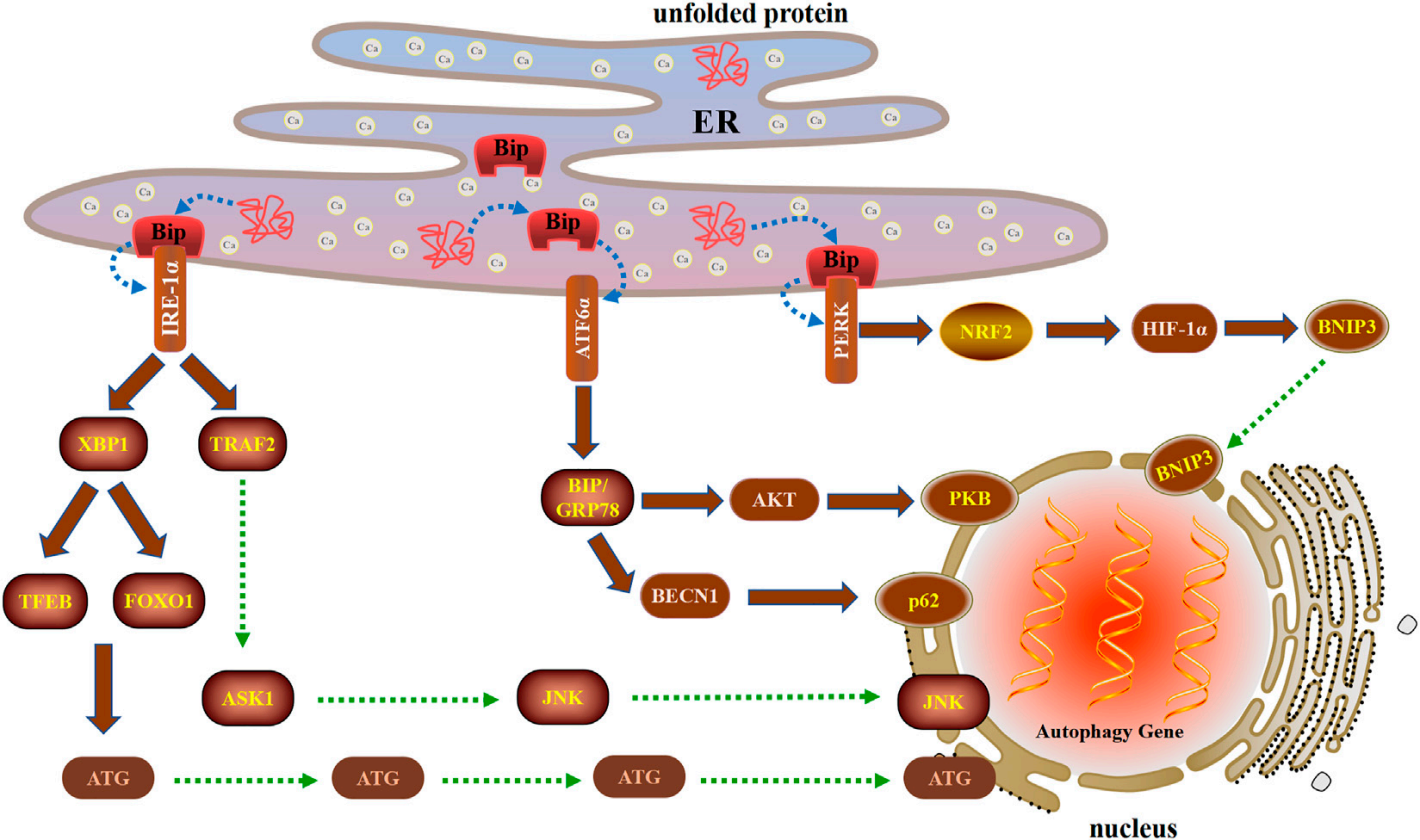
Three unique ways in which the UPR regulates autophagy during ERS after cell injury. The UPR is initiated by three ER transmembrane proteins: IRE1α, PERK, and ATF6α. IRE1α can induce autophagy by activating XBP1 and TRAF2. When ERS triggers the UPR, PERK is activated and then induces autophagy through the PERK-NRF2-HIF-1α-BNIP3 signaling pathway. ATF6α induces autophagy by upregulating the expression of BIP and inhibiting the AKT and BECN1 signaling pathways.

During ERS, cells use an integrated signaling system to restore homeostasis and normal ER function. The basic pathways of this system include the unfolded protein response (UPR), ER-associated degradation, autophagy, hypoxia signal transduction, and mitochondrial biogenesis (Senft and Ronai, 2015). Autophagy is strictly regulated by the protective mechanism of the UPR. The UPR and autophagy are interrelated, indicating that ERS both induces and suppresses autophagy. The UPR is initiated by three ER transmembrane proteins: inositol-requiring 1α (IRE1α), PERK, and activating transcription factor 6α (ATF6α). In recent years, UPR has the latest way to regulate autophagy based on the above three unique ways during ERS (Figure 2).

#### 2.2.1 UPR-IRE1-XBP1-TFEB/FOXO1 autophagy

The X-box binding protein 1 (XBP1) is not only an important component of the UPR, but also an important nuclear transcription factor. XBP1 is a member of the CEB/P family of transcription factors that bind to UPR elements (UPRE) on their target genes (Lee et al., 2003). XBP1 is the downstream target of IRE1α. Under the ERS, XBP1 is spliced by IRE1α, resulting in functional spliced XBP1 (XBP1s). XBP1s initiates transcription programs by translocation into the nucleus, which regulate UPR involved in the pathophysiology of various diseases (Wu et al., 2015). XBP1s occupies the −743 to −523 site of the promoter of TFEB, a major regulator of autophagy and lysosomal biogenesis. In mouse liver, the deletion of XBP1 inhibited the transcription of TFEB and autophagy, while the overexpression of XBP1s enhanced TFEB transcription as well as autophagy (Zhang et al., 2021). Furthermore, XBP1 regulated autophagy through the interaction of FOXO1 under ERS condition (Kishino et al, 2017). In the low oxidative stress group, FOXO1 was exported from the nucleus, modified into Ac-FOXO1 and bound to ATG7 in the cytoplasm, thereby promoting autophagy to protect cells. In the high oxidative stress group, FOXO1 was located in the nucleus, promoted the transcription of proapoptotic proteins, and then induced apoptosis (Lu et al., 2021). Therefore, FOXO1 can be used as a redox sensor switch to regulate the transformation of autophagy and apoptosis.

#### 2.2.2 UPR-IRE1-TRAF2-ASK1-JNK autophagy

The cytoplasmic part of IRE1 bound tumor necrosis factor receptor-associated factor 2 (TRAF2), an adaptor protein that couples plasma membrane receptors to JNK activation (Urano et al., 2000). IRE1 participates in the activation of JNK by forming TRAF2-ASK1 complex, thereby inducing the formation of autophagy, which required BECN1 transcription by c-Jun (Liu et al., 2020b). High glucose and bupivacaine induced cytotoxicity in SH-SY5Y cells through enhancing cell apoptosis and inhibiting autophagy via the IRE1-TRAF2 signaling pathways (Liu et al., 2019a). CHIP (carboxyl terminus of HSC70-interacting protein) mediated ubiquitination of IRE1 contributes to the dynamic regulation of UPR, which requires the involvement of IRE1/TRAF2/JNK pathway (Zhu et al., 2014). The ketamine enhanced autophagy and ERS *in vivo* and *in vitro* via restraining IRE1/TRAF2/ASK1/JNK pathway (Yu et al., 2021). In addition, Wogonoside exhibited antitumor activity by inducing ERS-associated autophagy and cell death by the IRE1α/TRAF2/ASK1 pathway (Gu et al., 2021).

#### 2.2.3 UPR-PERK-NRF2-HIF-1α-BNIP3 autophagy

When ERS triggers the UPR, the PERK signaling pathway is activated upon PERK dimerization and autophosphorylation (Walter et al., 2015). The nuclear factor-erythroid-2-related factor 2 (NRF2) is the main regulator of HIF-1α and can be activated as a downstream effector of PERK signaling (Ma et al., 2018). In addition, HIF-1α is considered to be the key effector of the cellular response to hypoxia, while NRF2 is the main antioxidant transcription factor and the key factor essential for HIF-1α-mediated hypoxia response (Mahajan and Sitasawad, 2021). Autophagy induced by HIF-1α/BNIP3 signaling pathway plays a protective role in myocardial ischemia-reperfusion injury (Zhang et al., 2019c). In the neonatal corpus luteum, HIF-1α up regulates BNIP3 expression, promotes the initiation of autophagy by disrupting BECN1 of BCL-2/BECN1 complex, and protects cells from apoptosis by curbing the skew of mitochondria balance under avascular niche (Tang et al., 2020). Berberine mediates BNIP3 expression by enhancing the binding of HIF-1α to the BNIP3 promoter, thereby inducing cardiomyocyte proliferation and autophagosome formation to inhibit cardiomyocyte apoptosis and myocardial ischemia/reperfusion injury (Zhu et al., 2020).

#### 2.2.4 UPR-ATF6α-BIP/GRP78-AKT/BECN1 autophagy

The ATF6α branch of the UPR is the least understood aspect of ERS and autophagy. ATF6α primarily regulates the expression of ER resident proteins involved in the maturation and degradation of ER client proteins (Bommiasamy et al., 2009). The binding immunoglobulin protein (BIP)/glucose-regulated protein 78 (GRP78) through ATF6α regulation increases the folding capacity of ER protein, contributing to resolution of the stress, and helps reverse UPR activation (Yu et al., 2016). ATF6α participates in the initiation of autophagy by up-regulating the expression of the BIP and inhibiting the expression of PKB of the AKT1/AKT pathway (Yung et al., 2011). Interestingly, crosstalk between apoptosis and autophagy can be regulated by the arginylated BIP/BECN1/p62 complex. Dephosphorylation of BECN1 increases the cleavage of BECN1 and destroys the BIP/BECN1/p62 complex, which leads to autophagy to the synergistic induction of apoptosis (Song et al., 2018).

With the discovery of UPR components and the elucidation of its mechanism, many studies have shown that its signaling pathway plays an important role in the process of nervous system diseases. By establishing blast-induced traumatic brain injury, administration of the ERS inhibitor salubrinal (SAL) found that SAL reduced CHOP protein expression and diminished Caspase-3 cleavage, suggesting attenuation of apoptosis. Interestingly, SAL also ameliorated impulsive-like behavior indicative of head trauma (Logsdon et al., 2014). The Neuro-2a cells and primary cultured mice cortical neurons were subjected to oxygen-glucose deprivation and reperfusion (OGD/R). Urolithin A (Uro-A) alleviated OGD/R-induced injury in Neuro-2a cells and neurons and reduced ischemic brain injury in mice. And Uro-A reinforced ischemia-induced autophagy. Furthermore, Uro-A-conferred protection was abolished by 3-methyladenine, suggesting the requirement of autophagy for neuroprotection (Ahsan et al., 2019). Exposure of Neuro-2a cells to MeHg also triggered ERS. Pretreatment with the antioxidant N-acetylcysteine (NAC) effectively prevented MeHg-induced neuronal cell ROS generation, apoptotic and ERS-related signals, and Akt inactivation (Chung et al., 2019a). These observations provide evidence UPR is a promising molecular target for the attenuation of neuronal injury.

### 2.3 Autophagy and mitochondrial dysfunction

Abnormal or dysfunctional mitochondria are cleared via selective autophagy, a process termed mitophagy (Ney, 2015). In addition to playing an indispensable role in the renewal of normal mitochondria, mitophagy prevents the accumulation of cytotoxic byproducts resulting from mitochondrial damage. Impaired or insufficient mitophagy leads to aggregation of damaged mitochondria, resulting in an uncontrolled increase in ROS production, mitochondrial DNA mutation, and energy failure, ultimately leading to cell death.

Neuronal cell death in brain disorders (neurodegeneration) and injury (neurotoxicity and ischemia) has been linked to a variety of alterations in mitochondrial homeostasis/function including traffic, quality control and turnover, homeostasis (bioenergetics and electron transport) and signaling (metabolism and Ca^2+^ handling) (Yin et al., 2014). Mitochondrial dysfunction with the concomitant energy failure and increased generation of ROS are considered central to neuronal cell loss in brain disorders because neurons have a limited capacity to upregulate glycolysis or to counteract oxidative damage (Markaki and Tavernarakis, 2020). Sequestration of mitochondria in autophagosomes occurs through three different cellular mechanisms, which are known as type 1, type 2, and type 3 mitophagy, each with its own characteristics (Table 1).

**TABLE 1:** Characteristics of the three types of mitochondria.

Types of mitophagy	Type 1	Type 2	Type 3
PI3K	Yes	No	No
BECN1	Yes	No	No
ULK1	Yes	No	No
LC3	Yes	Yes	No
PINK1	No	Yes	Yes
Parkin	No	Yes	Yes
MFN2	No	Yes	Yes
SQSTM1	No	Yes	No
p62	No	Yes	No

#### 2.3.1 Type 1 mitophagy

In type 1 mitophagy, PI3K, BECN1, ULK1, and other proteins for phagocytes along with LC3, the mammalian ortholog of yeast ATG8 (Li et al., 2011; Lemasters, 2014; Yamano and Youle, 2020). Phagocyte formation involves the growth of small pre-autophagic structures (PAS) containing LC3 associated with mitochondria. PAS expand to surround and sequester individual mitochondria into mitophagosomes, which requires the participation of PI3K and usually occurs in conjunction with mitochondrial fission (Lemasters and Zhong, 2018). After sequestration is completed, the outer compartment of the mitophagosome (the space between the inner and outer autophagosomal membranes) is acidified, followed by depolarization of the mitochondria. Finally, mitochondrial phagosomes containing depolarized mitochondria fuse with lysosomes (or late endosomes) to form autolysosomes, in which hydrolytic digestion of mitochondria occurs (Lemasters, 2014). Mitophagy induced by nutrient deprivation is a typical representative of type 1 mitophagy, and this is independent of PTEN-induced putative kinase 1 (PINK1) and parkin (an E3 ligase). The formation of autophagosomes during type 1 mitophagy is inhibited by PI3K inhibitors, such as wortmannin and 3-methyladenine (Dombi et al., 2018).

#### 2.3.2 Type 2 mitophagy

Mitophagy induced by photodamage is called type 2 mitophagy and involves PINK1 and parkin but does not require PI3K (Dombi et al., 2018). PINK1 requires depolarization of the mitochondrial membrane potential to pass through the inner mitochondrial membrane. Under normal conditions, PINK1 is cleaved in the transmembrane segment by the mitochondrial intramembrane protease presenilin-associated rhomboid-like and then released into the cytosol for proteasomal degradation (Jin et al., 2010). Parkin assembles the ubiquitin chain on the MOM and also recruits more parkin from MOM to amplify the “phospho-ubiquitin” mitophagy signal produced by PINK1 (Kane et al., 2014). Mitofusin 2 (MFN2) phosphorylated by PINK1 also promotes parkin recruitment to generate ubiquitin chains on the MOM and initiate mitophagy (McLelland et al., 2018). MFN2 is required for adaptation of mitochondrial respiration to stress conditions and produce ROS. In the absence of MFN2, insufficient ROS production can affect cytokine activity and induce the production of NO, which is related to dysfunctions in autophagy, apoptosis, phagocytosis, and antigen processing (Lloberas et al., 2020).

Unlike type 1 mitochondrial autophagy, goblet phagocytes are not formed during type 2 mitochondrial autophagy, and mitochondrial fission is not obvious (Lemasters, 2014). Damaged mitochondria accumulate PINK1 and then recruit parkin, leading to ubiquitination of mitochondrial proteins (Ivankovic et al., 2016). These ubiquitinated proteins are then sequestered by the autophagy proteins sequestosome 1 (SQSTM1)/p62 and LC3, ultimately leading to the degradation of the mitochondria via mitophagy (Lamark et al., 2017). SQSTM1/p62 is a stress-induced cellular protein complex that functions as a selective autophagy receptor to degrade ubiquitinated substrates (Katsuragi et al., 2015). The ZZ-type zinc finger domain of SQSTM1/p62 (SQSTM1/p62_ZZ_) recognizes a degradation signal in proteins (an N-terminal arginine residue). Recognizing the internal SQSTM1/p62 sequence through SQSTM1/p62_ZZ_ modulates the activity of SQSTM1/p62 through post-translational modification (Zhang et al., 2019d). In addition, PI3K inhibitors cannot block the type 2 mitophagy (Lemasters and Zhong, 2018).

#### 2.3.3 Type 3 mitophagy (mitochondria-derived vesicles [MDVs] or micromitophagy)

The third type of mitochondrial autophagy involves the formation of MDVs that germinate from mitochondria and then transfer to lysosomes (Soubannier et al., 2012a). MDVs are cargo-selective vesicles released from mitochondria independent of the mitochondrial fission mechanism. Oxidative stress stimulates the formation of MDVs, which are rich in oxidized mitochondrial proteins (Vasam et al., 2021). MDVs respond early to oxidative stress, the degree of which is determined by the respiratory state of the mitochondria. Unlike type 2 mitophagy, the formation of MDVs does not require mitochondrial depolarization. Although the formation and transport of MDVs to lysosomes do not involve the autophagy proteins ATG5 and LC3 (Soubannier et al., 2012b), PINK1 and parkin are required (Roberts et al., 2016). During the import process, PINK1 is first cleaved by matrix processing peptidase and presenilin associated rhomboid-like protein, and then cleaved PINK1 is released from the input channel and degraded in the cytoplasm via the N-end rule proteolytic pathway. After the mitochondria are depolarized, the import mechanism is inactivated, and PINK1 is trapped in the import channel or anchored on the MOM near the import channel. This exposes the kinase domain to the cytosol, where it phosphorylates ubiquitinates parkin, resulting in the recruitment and activation of stable parkin on the mitochondrial surface. Parkin then ubiquitinates a series of proteins on the surface of mitochondria, which are then recognized by autophagy adaptor proteins and delivered to autophagosomes (Greene et al., 2012; Lazarou et al., 2012; Sarraf et al., 2013; Yamano and Youle, 2013; Kazlauskaite et al., 2014).

In addition to the above signaling pathways, NIX/BNIP3L, FUNDC1 and p62/SQSTM1 can also mediate mitophagy. The most well-accepted mechanism underlying BNIP3L-mediated mitophagy is through an interaction with proteins from the Atg8 family, by which BNIP3L recruits autophagosomes to targeted mitochondria (Novak et al., 2010). Moreover, BNIP3L disrupts the BCL2-BECN1 complex and releases BECN1, which promotes the formation of autophagosomes (Mazure and Pouysségur, 2009). FUNDC1 is a mammalian mitophagy receptor that interacts with and recruits LC3 to mitochondria for mitophagy (Liu et al., 2012). FUNDC1 is phosphorylated at tyrosine 18 (Y18) and serine 13 (S13) by SRC kinase and CK2, respectively. The phosphorylation prevents the interaction between FUNDC1 and LC3 for subsequent mitophagy in a mammalian system (Chen et al., 2016). p62/SQSTM1, a cytoprotein, containing a cytosolute LIR motif protein, was the first mammalian selective autophagy receptor to be described. In addition to the above receptor proteins, other cytoplasmic receptor proteins, such as NBR1, NDP52, TANK-Binding Kinase1, and Optineurin, have also been confirmed to mediate the mitophagy (Heo et al., 2015; Shi et al., 2015; Vargas et al., 2019; Di Rita et al., 2021).

### 2.4 ER and mitochondrial dysfunction

ER-mitochondria (ER-mito) contact sites (MERCs) are morpho-functional units, formed at the loci of close apposition of the ER-forming endomembrane and outer mitochondrial membrane (OMM) and mitochondria-associated membrane (MAM). These sites contribute to fundamental cellular processes including lipid biosynthesis, autophagy, apoptosis, ERS and calcium (Ca^2+^) signaling (Lim et al., 2021). Mitochondrial Rho GTPase 1 (Miro1) protein is a well-known adaptor for mitochondrial transport and also regulates mitochondrial quality control and function. Furthermore, Miro1 was associated with MERCs, which are key regulators of cellular calcium homeostasis and the initiation of autophagy. The R272Q mutation in Miro1 not only enhances mitochondria and ER tethering, but also interferes with the function of MERCs in regulating calcium homeostasis and initiation of mitophagy (Berenguer-Escuder et al., 2020). Mitofusin2 (Mfn2) is widely accepted as an essential molecule involved in ER-mito juxtaposition (Han et al., 2021). Mul1–Mfn2 pathway maintains neuronal mitochondrial integrity through its dual-role in regulating mitochondrial morphology and ER-Mito contacts. This mechanism ensures that mitophagy degradation is restrained in neurons under early stress conditions (Puri et al., 2019). In non-neuronal cell lines, Mul1 acts as an E3 ubiquitin ligase that binds, ubiquitinates, and degrades Mfn2 and as a small ubiquitin-like modifier (SUMO) E3 ligase that SUMOylates dynamin-related protein-1 (Drp1) to enhance its stability on mitochondria (Prudent et al., 2015).

The mitochondrial contact site and cristae organization system (MICOS) is a multi-subunit complex present in the inner mitochondrial membrane. The Mic60 and Mic10 subcomplexes are the core MICOS protein subcomplexes and associate together with other peripheral proteins and protein complexes to form the mature MICOS complex. Mic60 appears to be the core subunit. Its loss decreases other MICOS subunits and is detrimental to cristae architecture, leading to a variety of observed abnormal mitochondrial phenotypes including concentric cristae rings, swirls or onion-like shapes, detachment of cristae from the OMM, a loss of cristae and disconnected cristae tubules. These cristae abnormalities compromise the respiratory capacity of the cell, leading to decreased cellular proliferation and survival. Mic10 also exists in a subcomplex and while its loss does not affect the assembly of the Mic60 subcomplex, characteristic cristae abnormalities associated with a disrupted MICOS are observed (Eramo et al., 2020). Mic19 is directly linked to Mic60, forming a subcomplex that can be separated from Mic10-based subcomplexes, which assembles independent of respiratory complexes and the mitochondrial lipid cardiolipin (Friedman et al., 2015).

## 3 Autophagy and nervous system diseases

**FIGURE 3:**
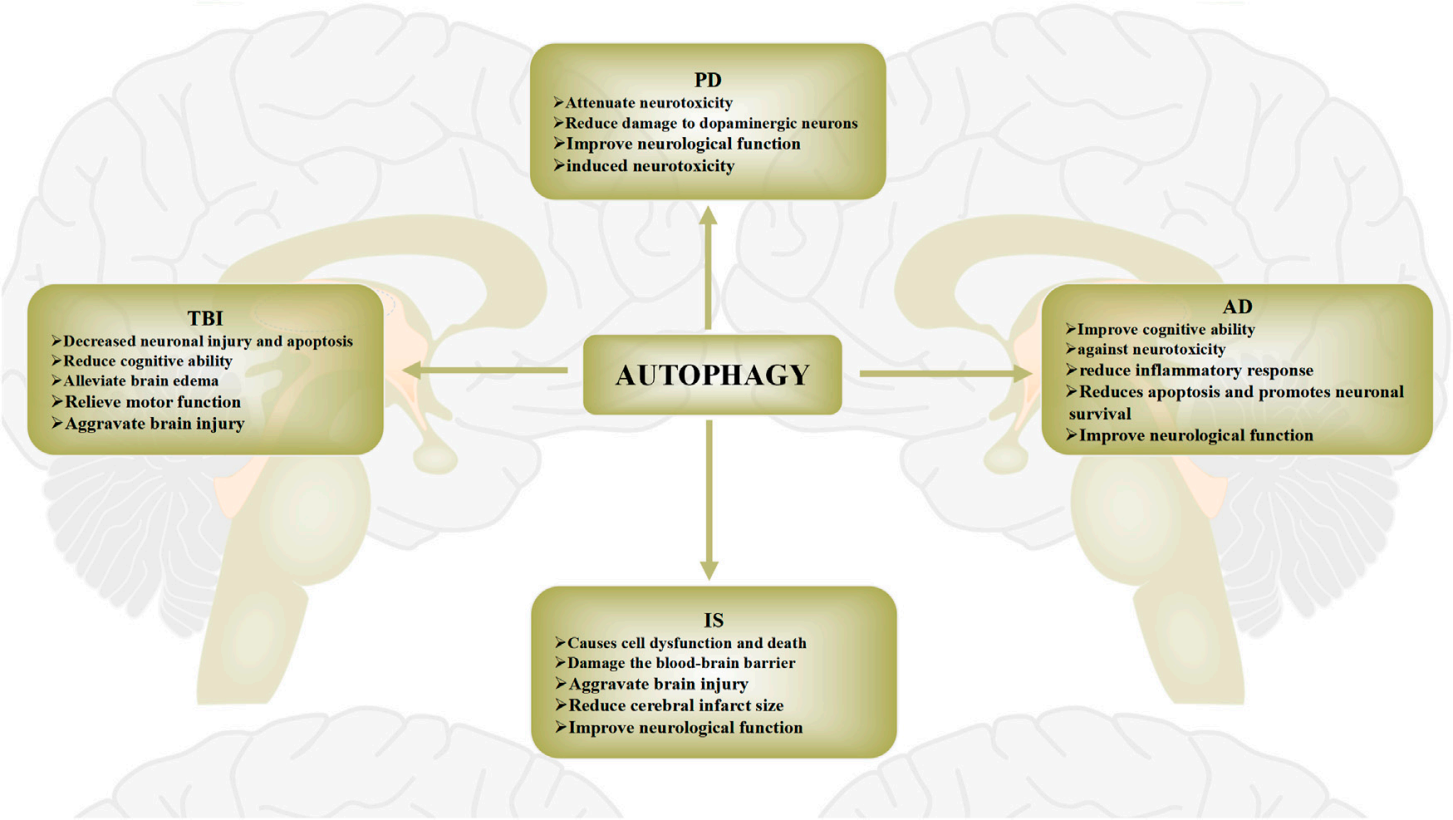
Role of autophagy in brain injury diseases. The role of autophagy in brain injury diseases has recently been widely reported. Protective and pathogenic roles of autophagy in these diseases have been proposed. The role of autophagy in brain injury diseases is widely regarded as a “double-edged sword”. Autophagy activation after brain injury removes necrotic substances, thereby preventing cerebral ischemia damage and promoting cell survival. Conversely, activation of autophagy may also further aggravate brain damage, leading to cell death.

Autophagy plays an important role in central nervous system diseases through a variety of mechanisms, including the elimination of damaged organelles, prevention of protein aggregate accumulation, and maintenance of basic cell homeostasis (Bingol, 2018). As the various physiological and pathological mechanisms of autophagy have been elucidated, many studies exploring the role of autophagy in neurological diseases have also been reported (Bar-Yosef et al., 2019). In this section, we will discuss the mechanism of autophagy in traumatic brain injury (TBI), intracerebral hemorrhage (IS), Alzheimer’s disease (AD), Parkinson’s disease (PD), and Huntington’s disease (HD), and Amyotrophic lateral sclerosis (ALS) (Figure 3).

### 3.1 Autophagy and TBI

TBI is an acquired injury to the brain caused by external mechanical forces that can result in temporary or permanent impairment (Capizzi et al., 2020). The symptoms of TBI vary from mild alterations of consciousness to an unrelenting comatose state and death. In the most severe cases of TBI, the entirety of the brain is affected by a diffuse type of injury and swelling (Galgano et al., 2017). TBI is the main cause of morbidity and mortality worldwide (Najem et al., 2018). A growing body of experimental and clinical evidence indicates that alterations in autophagy-related proteins are widespread after TBI. The pathogenesis of TBI is related to the up-regulation of autophagy flux. The most common mechanisms include an increase in BECN1 protein levels and decreased formation of BECN1/BCL2 complexes (Diskin et al., 2005). LC3 and BECN1 protein levels begin to increase in neurons 1 h after TBI and increase in astrocytes after 3 days, peaking 8 days later (Zhang et al., 2008). Liu et al. (Liu et al., 2020c) found that compared with healthy individuals, the expression of receptor-interacting protein kinase 1 (Ripk1), NF-κB, and NF-kB inhibitor α in the blood of TBI patients is significantly up-regulated. After silencing of Ripk1 or inhibition of the NF-κB signaling pathway, the expression of interleukin (IL)-1β, IL-6, TNF-α, Bax, and caspase-3 is down-regulated, whereas that of BCL-2, ATG5, and LC3II/LC3I is up-regulated. In addition, the down-regulation of Ripk1 significantly reduces neuronal damage and apoptosis and significantly increases neuronal autophagy. Overexpression of Ripk1 contributes to activation of the NF-κB signaling pathway, thereby aggravating the damage induced by TBI. Silencing Ripk1 suppresses the effects of TBI by inhibiting the NF-κB signaling pathway and promoting neuronal autophagy. Luo et al. (Luo et al., 2011) reported increases in LC3, BECN1, cathepsin B, and caspase-3 expression and the BECN1/BCL2 ratio after TBI.

Autophagy is associated with secondary injury following TBI, suggesting autophagy could be a therapeutic target. In a moderate TBI model, activation of autophagy was shown to exert a neuroprotective effect by inhibiting pyroptotic cell death in a process dependent on the down-regulation of IL-13 and repression of the JAK-1–STAT-1 signaling pathway (Gao et al., 2020a). Exogenous calcitonin gene–related peptide reduces apoptosis and autophagy after TBI via the AKT/mTOR signaling pathway and alleviates cognitive decline in TBI mice, thereby exerting a neuroprotective effect (Tian et al., 2020). In a microvascular endothelial cell model of TBI, hydrogen increased cell viability by inhibiting autophagy via activation of the PI3K/AKT/glycogen synthase kinase-3beta (GSK3β) signaling pathway (Wang et al., 2020a). Post-treatment with sevoflurane (He et al., 2018) and tetrahydrocurcumin (Gao et al., 2016) regulates autophagy via the PI3K/AKT signaling pathway, attenuating TBI-induced neuronal apoptosis. Activation of the IL-33/ST2L (suppression of tumorigenicity 2 receptor) signaling pathway prevents increases in IL-1β and TNF-α levels induced by TBI, inhibiting the up-regulation of autophagy after TBI and alleviating TBI-induced brain edema, motor function effects, and spatial learning and memory defects (Gao et al., 2018).

### 3.2 Autophagy and IS

IS a common cerebrovascular disease caused by a sudden blockage of a blood vessel in the brain that limits the blood supply to the area involved (Wang et al., 2021b), ultimately leading to brain injury and neurological dysfunction (Akella et al., 2019). IS has high mortality and disability rates globally (Datta et al., 2020). Autophagy is involved in the pathogenesis of IS, and modulating autophagy activity can alter the prognosis of IS. However, whether IS-associated autophagy is beneficial or detrimental to neuronal cell survival is unclear and a matter of debate. After IS, autophagy is activated by various cell types in the brain, such as neurons, glial cells, and vascular endothelial cells (Zhang et al., 2020c), which leads to the removal of necrotic substances resulting from cerebral ischemic injury, thereby promoting cell survival. Conversely, activation of autophagy can also further aggravate ischemic brain injury, leading to cell death. Autophagy has been well characterized as a therapeutic target for IS (Wang et al., 2020b).

#### 3.2.1 Detrimental role of autophagy in IS

The results of *in vivo* and *in vitro* studies suggest that autophagy adversely affects the outcome of IS. Astrocyte-derived exosomes ameliorate neuronal injury after IS by modulating autophagy to inhibit the expression of caspase-3 and Bax and modulate the levels of TNF-α, IL-6, and IL-1β (Pei et al., 2019). In a rat model of IS, activation of autophagy in brain endothelial cells was shown to mediate occludin degradation, resulting in disruption of the blood-brain barrier, ultimately leading to cellular dysfunction and death (Kim et al., 2020). IL-17A mediates hyperautophagy, which aggravates the ischemic injury of neurons after IS via the Src-PP2B-mTOR pathway, whereas cyclosporine A blocks the induction of hyperautophagy (Liu et al., 2019b). FK506-binding protein 5 induces autophagy by regulating the AKT/FOXO3 signaling pathway, which aggravates nerve damage after IS (Yu et al., 2020b). Vitexin inhibits the induction of autophagy in the middle cerebral artery via mechanistic targeting of the mTOR/ULK1 pathway, decreasing the expression of ULK1 and BECN1 and the expression rate of LC3II/LC3I, reversing autophagy-related dysfunction and alleviating the symptoms of IS (Jiang et al, 2018). Exogenous netrin-1 inhibits autophagy via the PI3K/mTOR pathway, alleviating ischemic brain tissue damage and enhancing hypoxic neuron activity (Tang et al., 2019). Moreover, multiple agents, including melatonin (Feng et al., 2017) and homocysteine (Wang et al., 2019), reportedly either exert neuroprotective effects by inhibiting autophagy or aggravate ischemic brain injury by activating autophagy.

#### 3.2.2 Beneficial role of autophagy in IS

Considerable evidence also indicates that autophagy may exert a neuroprotective effect in IS. Ginaton induces autophagy by activating the AMPK pathway, thereby maintaining mitochondrial homeostasis and inhibiting apoptosis, which protects against ischemic neuronal injury (Li et al., 2019a). Overexpression of PINK1 was able to activate the PINK1-Parkin pathway and rescue IS-induced behavioral disorders. PINK1 protects IS‐induced brain injury by promoting mitochondrial autophagy in microglia, which may be of value as a therapeutic target for IS treatment (Li et al., 2021). The mTOR inhibitor rapamycin significantly reduces mTOR activation and infarct volume by activating autophagy and inhibiting neuronal apoptosis, improving neural function after IS (Wu et al., 2018a). Inducing autophagy flux of astrocytes *in vitro* enhances neuronal viability and reduces neuronal apoptosis, which contributes to the endogenous post-IS neuroprotection and neural recovery mechanism (Liu et al., 2018). Various agents, including compound C (Jiang et al., 2015) and eugenol (Sun et al., 2020), reportedly exert either neuroprotective effects by activating autophagy or harmful effects by inhibiting autophagy.

### 3.3 Autophagy and AD

AD, the most common neurodegenerative disease, is characterized by β-amyloid (Aβ) deposition and Tau phosphorylation (Zhang and Zheng, 2019). The clinical symptoms are progressive memory loss caused by the abnormal accumulation of misfolded proteins in neuronal cells, resulting in a loss of cellular protein homeostasis (Di Meco et al., 2020). There is a growing consensus that autophagy may be a pathophysiological process involved in AD (Eshraghi et al., 2021). The metabolism of Aβ and Tau is critically affected by autophagy.

#### 3.3.1 Autophagy and Aβ

Aβ is produced by the beta-amyloid precursor protein (APP) during autophagic renewal of APP-rich organelles generated by autophagy and endocytosis (Nixon, 2007). In the early stage of AD, Aβ activates autophagy and is then degraded after transport from autophagosomes to lysosomes (Han et al., 2018), which in turn regulates Aβ accumulation and aggregate formation (Bharadwaj and Martins, 2020). Autophagy is the major cellular pathway for protein aggregate removal, and upregulation of autophagy represents a rational therapeutic strategy against neurotoxic Aβ overproduction. Activation of PPARA-mediated autophagy reduces AD-like pathology and cognitive decline in mouse models (Luo et al., 2020). Crocetin induces autophagy in neuronal cells via serine/threonine kinase 11/LKB1(live kinase B1)-mediated activation of the AMPK pathway, significantly reducing Aβ levels and neuroinflammation in the brain and improving memory function in mice (Wani et al., 2021). Reportedly, AMPK activation does not induce autophagy in cerebellar granule neurons or cultured SH-SY5Y cells or affect Aβ secretion (Benito-Cuesta et al., 2021). In addition, alborixin (an ionophore) also exhibits strong neuroprotective effects, inhibiting the PI3K/AKT pathway to induce autophagy, upregulating the levels of BECN1, ATG5, and ATG7 to increase lysosomal activity, and significantly reducing the levels of Aβ in microglia and primary neurons, thus preventing or slowing the progression of AD (Wani et al., 2019).

#### 3.3.2 Autophagy and Tau

Phosphorylation of tau is another pathological feature of AD. Although the ubiquitin-proteasome system is considered the main pathway for Tau degradation, autophagy is thought to be another efficient degradation pathway (Chung et al., 2019b). It has been demonstrated that p300/CBP (CREB binding protein) overactivation blocks autophagic flux and increases tau secretion in neurons. Conversely, inhibition of p300/CBP promotes increased autophagic flux, decreases tau secretion, and reduces tau transmission (Chen et al., 2020). Polyethylene glycol (PEG)-ceramide nanomicelles increase the ratio of LC3-II/LC3-I and reduce the level of p62 protein, thereby increasing autophagic flux in N2a cells and the degradation of overexpressed human tau protein. PEG-ceramide nanomicelles thus hold tremendous potential as AD drugs to induce autophagy and degrade tau protein (Gao et al., 2020b). Dihydrotanshinone I (DTSI) significantly inhibits mTOR phosphorylation via the AMPK/mTOR signaling pathway, thereby enhancing autophagy, increasing Aβ clearance, and reducing tau phosphorylation, which highlights the potential of DTSI in the treatment of AD (Bao et al., 2020). Neuroprotectin D1 (NPD1) was shown to enhance autophagic activity, downregulate Aβ expression, and control tau hyperphosphorylation in AD cell models, thereby reducing apoptosis and promoting neuronal survival. The neuroprotective effect of NPD1 may be due to inhibition of GSK-3β activation in neuronal cells (Dai et al., 2021). In an AD cell model, galangin treatment was shown to inhibit the expression of BECN1 and p-GSK3β but promote autophagy and decrease levels of Aβ and p-Tau by regulating the AKT/GSK3β/mTOR pathway. Thus galangin is considered a potential drug candidate for the treatment of AD (Huang et al., 2019).

### 3.4 Autophagy and PD

PD is a neurodegenerative disease characterized by motor system dysfunction, with resting tremor, bradykinesia, rigidity, and postural instability as common clinical manifestations (Huang et al., 2019). The pathogenesis of PD includes multiple factors, such as autophagy (Cerri and Blandini, 2019), oxidative stress (Trist et al., 2019), mitochondrial dysfunction (Borsche et al., 2021), and impaired protein homeostasis (Masato et al., 2019). Autophagy, as a major degradation pathway, plays a key role in maintaining the efficient turnover of damaged proteins and organelles in cells. The accumulation of α-Syn and the degeneration of dopaminergic neurons are closely related to autophagy and represent two major features of the pathology of PD (Hou et al., 2020). α-Syn fibrils recruit selective autophagy-associated proteins TANK-binding kinase 1 and optineurin to sites of lysosomal damage and induce autophagy in microglial cells (Bussi et al., 2018). Overexpression of α-Syn activates CMA by increasing levels of LAMP-2A, whereas protein deglycase DJ-1 (Parkinson disease protein 7) promotes the activation of CMA, upregulates the expression of LAMP-2A and lysosomal HSC70, slows the degradation of LAMP-2A in lysosomes, and inhibits the aggregation of α-Syn (Xu et al., 2017). Enhanced expression of miR-19a-3p in exosomes from α-Syn transgenic SH-SY5Y cells inhibits autophagy in microglia by targeting the phosphatase and tensin homolog/AKT/mTOR signaling pathway. Overexpression of miR-7 promotes autophagy by targeting the 3′-untranslated region of its mRNA, resulting in the degradation of α-Syn and its aggregates and demonstrating that miR-7 is an attractive target for therapeutic intervention in PD (Choi et al., 2018). However, overexpression of miR-101 results in a significant increase in α-Syn accumulation and autophagy defects (Valera et al., 2017).

Crosstalk between autophagy dysfunction and dopamine neuron injury is closely related to the pathogenesis of PD. Following dopamine neuron damage in PD, microtubule-associated LC3-II and α-Syn are significantly up-regulated and p62 down-regulated, whereas insulin-like growth factor-1 (IGF-1) inhibits autophagy via the IGF-1R/PI3K-AKT-mTOR pathway, thereby attenuating neurotoxicity (Wang et al., 2020c). In a PD model, 6-hydroxydopamine/ascorbic acid (6-OHDA/AA) was shown to activate autophagy and induce neurotoxicity via the mucolipin 1/calcium/calcineurin/TFEB pathway. Interestingly, overexpression of TFEB attenuated the neurotoxicity of 6-OHDA/AA (Zhuang et al., 2020). The degradation mediated by autophagy also plays a positive role in the treatment of PD. Administration of curcumin enhances autophagy and increases the number of surviving dopamine neurons and expression of TFEB, LAMP-2A, and LC3-II proteins and decreases α-Syn protein and mRNA expression, which exerts a protective effect on PD dopamine neurons (Wu et al., 2018b). Salidroside exerts neuroprotective effects by enhancing PINK1/parkin-mediated mitophagy to protect dopaminergic neurons (Li and Chen, 2019). The Kir6.1/K-ATP channel on astrocytes also protects against dopaminergic neurodegeneration in PD by promoting mitophagy (Hu et al., 2019). Small-molecule enhancer of rapamycin 28 (an autophagy inducer) induces autophagy by increasing the activity of SOD and NRF2, reducing the production of ROS and the expression of p62, thereby alleviating the damage to dopaminergic neurons in the substantia nigra pars compacta of the brainstem and improving motor function (Darabi et al., 2018).

### 3.5 Autophagy and HD

Huntington’s disease (HD) is caused by a mutant huntingtin (mHTT) protein that contains abnormally extended polyglutamine (polyQ) repeats. The majority of neurodegenerative disorders, including HD, have a similar pathophysiology of progressive buildup of harmful protein aggregates. As neurons are terminally differentiated cells, efficient waste clearance is essential to prevent the formation of these build-ups. mHTT readily forms aggregates that make their degradation via the proteasome highly challenging due to its narrow entry, and autophagy thus functions as a major defender against mHTT aggregates. MicroRNAs (miRNAs) are new players in regulating autophagy. The extended polyQ in mouse striatal neurons increased lysosomal membrane-associated protein 2A (LAMP2A) levels and influenced the inflammatory conditions, and these augmented levels correlated to the let7b miRNA expression level. The upregulated let7b increased LAMP2A and reduced the extended polyQ in mouse striatal cells. The let7b level was highly expressed in the striatum of pre-onset HD mice, whereas it was significantly reduced in the post-onset HD striatum. These results suggest that LAMP2A related to CMA capacity might play an essential role in HD symptom onset and progression (Choi and Cho, 2021). FKBP5 is identified as a protein involved in the pathogenesis of HD. Reducing the level or activity of FKBP5 will lead to increased autophagy and decreased mHTT content. The structural changes of HTT protein may be due to changes in proline sites in the protein and subsequent protein clearance. Whereas rapamycin interacts with FKBP1A/FKBP12 and FKBP5/FKBP51to inhibit the mTORC1 complex and increase the cellular clearance mechanisms. This identified FKBP5 as a potential therapeutic target for HD (Bailus et al., 2021). Moreover, linker molecules that interact with both mHTT and LC3 can increase the clearance of mHTT and reduce pathology in cellular and animal models of HD (Li et al., 2019b).

### 3.6 Autophagy and ALS

ALS is a disease of protein dyshomeostasis, with autophagy dysfunction playing an essential role in the pathogenesis of ALS. Multiple ALS-linked genes, such as SQSTM1, OPTN, and TBK1, encode for core autophagy proteins and others, such as C9orf72, FUS, TDP43, VAPB, UBQLN2, VCP, CHMP2B, ALS2, FIG4, TUBA4A, PFN1, and DCTN have a functional role in autophagy (Amin et al., 2020). Moreover, the misfolded or aggregated protein products of some ALS-causing genes that are not directly involved in the autophagy process, such as SOD1, can abnormally interact with autophagy proteins to dysregulate their activity (Abati et al., 2020). Proteotoxic stress activates serine/threonine kinase TBK1, which coordinates with autophagy kinase ULK1 to promote concerted phosphorylation of autophagy receptor SQSTM1 at the ubiquitin-associated domain and activation of selective autophagy. In contrast, ALS-FTLD-linked mutations of TBK1 or SQSTM1 reduce SQSTM1 phosphorylation and compromise ubiquitinated cargo binding and clearance. Moreover, disease mutation SQSTM1G427R abolishes phosphorylation of Ser351 and impairs KEAP1-SQSTM1 interaction, thus diminishing NFE2L2/Nrf2-targeted gene expression and increasing TARDBP/TDP-43 associated stress granule formation under oxidative stress. Furthermore, expression of SQSTM1G427R in neurons impairs dendrite morphology and KEAP1-NFE2L2 signaling (Deng et al., 2020).

### 3.7 Autophagy and Post–traumatic neurodegeneration

TBI increases the likelihood of developing dementia, especially AD. It is becoming more and more clear how trauma might lead to neurodegeneration. There are several potential mechanisms by which TBI may increase dementia risk, because TBI can involve a variety of neural changes, depending on the nature and severity of the injury as well as the cumulative number of TBI an individual has experienced. Diffuse axonal and vascular damage can arise from closed head injury because the brain is subjected to forces of acceleration and deceleration that can strain and tear blood vessels and axons (Blennow et al., 2012). TBI-induced neural changes may serve to lower individuals’ cognitive reserve and thus increase their propensity to exhibit cognitive decline in older adulthood, particularly when brain changes associated with normal aging start to occur in a neural system that has already undergone damage (Barnes et al., 2014). Additionally, neurodegeneration is influenced by the control of autophagy, a cellular homeostatic mechanism that targets, breaks down, and recycles protein aggregates and cellular organelles. Morin post-treatment confers neuroprotection in a novel rat model of mild repetitive traumatic brain injury by targeting dementia markers, APOE, autophagy and Wnt/β-catenin signaling pathway. Where Morin alone and when combined with MK-801 abated the dementia surrogate markers (Aβ42, p (Thr 231) Tau, APO-E, p (Ser33) β-catenin) and enhanced the autophagy process (LC3BII/I ratio, Beclin-1). Besides, it reduced inflammatory markers (p (Ser536) NF-κBp65, TNF-α, IL-6) and modulated the apoptotic/anti-apoptotic parameters (caspase-3, Bcl-2) (El-Gazar et al., 2019).

## 4 Discussion

This review highlights the key contribution of autophagy and its properties in the pathogenesis of brain injury. We provide an overview of autophagy and focus on the crosstalk between autophagy, oxidative stress, and ERS. The interaction between autophagy and ERS is involved in the regulation of the effects of brain injury. In short-term brain injury, autophagy and ERS play a protective role. In long-term brain injury, the ERS response is over-activated, and the degradation capacity of autophagy decreases, thus aggravating brain injury. Oxidative stress plays a major role in regulating cell death by affecting the ER and mitophagy. Oxidative stress/ROS production triggers ERS, which further increases the production of ROS. Further study is needed to clarify the important roles of autophagy, oxidative stress, and ERS in regulating brain injury and to clarify the regulatory pathways involved in autophagy and its regulatory role in brain injury. Such studies would provide useful theoretical guidance and identify potential new targets for the treatment of brain injury-related diseases.
